# Antioxidant Activity and α-Glucosidase Inhibitory Activities of the Polycondensate of Catechin with Glyoxylic Acid

**DOI:** 10.1371/journal.pone.0150412

**Published:** 2016-03-09

**Authors:** Sheng Geng, Sharui Shan, Hanjun Ma, Benguo Liu

**Affiliations:** 1 School of Food Science, Henan Institute of Science and Technology, Xinxiang, 453003, China; 2 Department of Rehabilitation, The First Affiliated Hospital/School of Clinical Medicine of G.D.P.D, Guangzhou, China; Islamic Azad University-Mashhad Branch, Mashhad, Iran, ISLAMIC REPUBLIC OF IRAN

## Abstract

In order to investigate polymeric flavonoids, the polycondensate of catechin with glyoxylic acid (PCG) was prepared and its chemically antioxidant, cellular antioxidant (CAA) and *α*-glucosidase inhibitory activities were evaluated. The DPPH and ABTS radical scavenging activities and antiproliferative effect of PCG were lower than those of catechin, while PCG had higher CAA activity than catechin. In addition, PCG had very high *α*-glucosidase inhibitory activities (IC_50_ value, 2.59 μg/mL) in comparison to catechin (IC_50_ value, 239.27 μg/mL). Inhibition kinetics suggested that both PCG and catechin demonstrated a mixture of noncompetitive and anticompetitive inhibition. The enhanced CAA and *α*-glucosidase inhibitor activities of PCG could be due to catechin polymerization enhancing the binding capacity to the cellular membrane and enzymes.

## Introduction

Flavonoids are widely distributed in plants and are reported to possess many beneficial bioactivities—including as antioxidant, anti-inflammatory, antihypertensive, antiviral, antihyperglycemic and antitumor activities—which can be applied to food, medical and cosmetic industries [1–3]. However, the bioactivities and practical applications of flavonoids are hindered by poor aqueous solubility and pro-oxidant activities [4–6], which could be resolved by polymerization [7]. There are many naturally-occurring flavonoid polymers in foods and medicinal plants, such as theaflavins, proanthocyanidins and tannins, have been reported to possess higher biological and pharmacological activities than flavonoid monomers [7, 8]. The flavonoid monomer also could be oligomerized by laccase [9]. Wine contains a condensate of catechin with acetaldehyde, which is related to both the flavor and color of red wine [10, 11] and, inspired by these reports, a series of catechin-aldehyde polycondensates have been synthesized in an ethanol:water mixture via reactions with various aldehydes [12]. In comparison to a catechin monomer, these polycondensates exhibit higher superoxide anion-scavenging and human low-density lipoprotein inhibitory activities [13], as well as outstanding inhibitory capacities towards xanthine oxidase and tyrosinase [7, 14].

Among these novel molecules, the polycondensate of catechin with glyoxylic acid (PCG) has high aqueous solubility and numerous carboxyl groups, which can be further modified. Today, there were few reports about polymeric flavonoids, which hindered their application in food and medical industries. To increase understanding of this kind of macromolecule, PCG was synthesized and evaluated for cellular antioxidant and α-glucosidase inhibitory activities.

## Materials and Methods

### Chemicals

Catechin and glyoxylic acid were purchased from Aladdin (Shanghai, China). Quercetin, 2,2′-azino-bis(3-ethylbenzothiazoline-6-sulfonic acid) diammonium salt (ABTS), 2,2-dipheny-l-picrylhydrazyl (DPPH), dichlorofluorescin diacetate (DCFH-DA) and 2, 2’-azobis-amidinopropane (AAPH), α-glucosidase (from *Saccharomyces cerevisiae*), and p-nitrophenyl-α-D-glucopyranoside (pNP-G) were obtained from Sigma (St. Louis, MO). HepG2 human liver cancer cells were obtained from the American Type Culture Collection (Rockville, MD). Williams’ medium E, heparin, insulin, and epidermal growth factor were purchased from Gibco U.S. Biotechnology Co. Ultrapure water from a GenPure UV/UF laboratory water system (Thermo Fisher Scientific Inc.) was used for all experiments.

### Preparation of PCG

**Fig 1:**
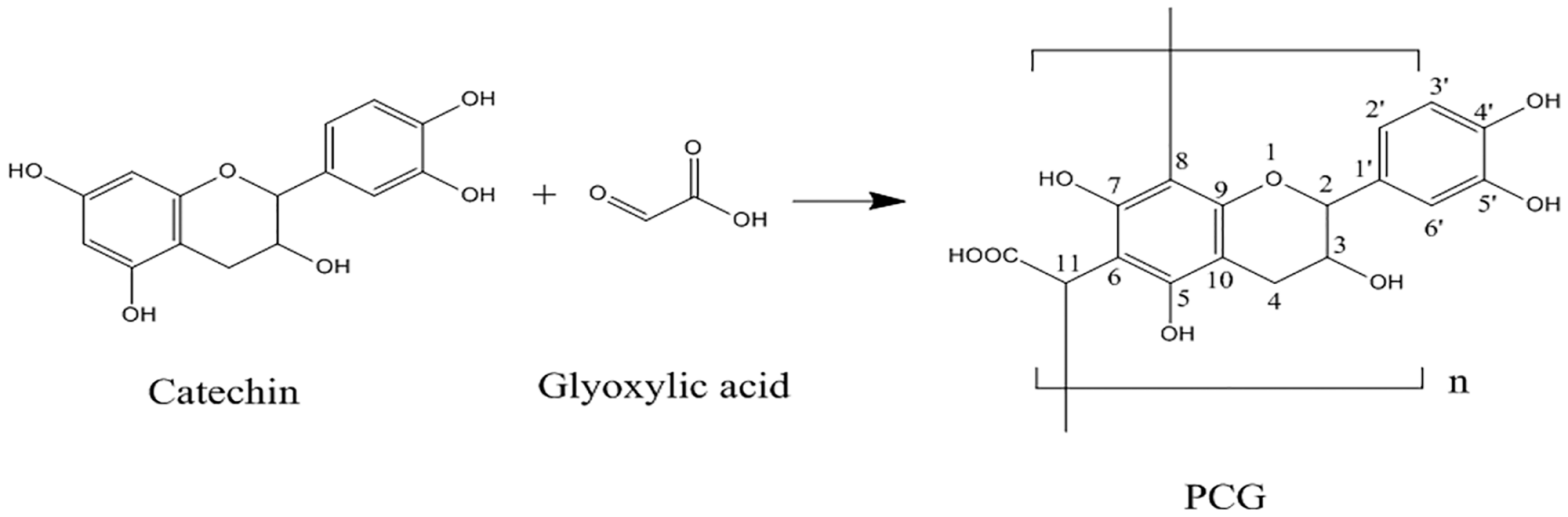
Synthetic mechanism of PCG.

PCG was synthesized as described previously with minor modifications (Fig 1) [12]. Catechin (0.37 g, 1.3 mM) and glyoxylic acid (2.99 g, 32.5 mM) were mixed in 25 mL of aqueous ethanol solution (12%, v/v) and stirred at 25°C for 24 h in the dark under vacuum, followed by filtration. The supernatant was dialyzed against the aqueous ethanol solution (12%, v/v) at room temperature for 24 h and then freeze-dried. The red powder (~150 mg) was identified as PCG and used for the following analysis.

### Structural Characterization

PCG was dissolved in methanol and UV spectra was measured using a TU-1810PC UV spectrophotometer (Purkinje, Beijing, China) over a scanning range of 220 to 400 nm. IR spectra were obtained on a TENSOR 27 FT-IR spectrophotometer (Bruker, Germany) using the KBr method. ^1^H and ^13^C NMR data were obtained using a 600 MHz Bruker NMR spectrometer at 25°C with DMSO-d_6_ as solvent.

### DPPH Radical Scavenging Assay

DPPH radical-scavenging activity of the sample was measured as previously described with minor modifications [15]. Briefly, 2 mL of DPPH solution (dissolved 0.1 mM in ethanol) was mixed with 2 mL of the sample in ethanol at different concentrations (2–20 μg/mL) and incubated in the dark at 25°C for 30 min and the absorbance was measured at 517 nm (A_sample_) was measured. The absorbance of a negative control (A_control_) composed of only ethanol was also determined. The DPPH radical scavenging activity of the sample was calculated by the following equation.

$$\text{DPPH scavenging activity }=\;\frac{{\text{A}}_{\text{control}}{\text{-A}}_{\text{sample}}}{{\text{A}}_{\text{control}}}\times \text{ 100\%}$$ (1)

### ABTS Radical Scavenging Assay

The ABTS radical scavenging activity of the sample was measured as previously described with minor modification [16]. 100 mL of 7 mM ABTS solution and 1.7 mL of 140 mM potassium persulfate solution were mixed together and incubated in the dark at 25°C for 12 h. The ABTS solution was diluted with phosphate buffer (0.05 M, pH 7.4) to obtain the working solution with an Abs_734_ of 0.70 ± 0.02. 150 μL of the sample dissolved in methanol at different concentrations (5–60 μg/mL) and mixed with 2.85 mL of working solution and incubated in the dark at 25°C for 10 min, and the absorbance of the sample at 734 nm (A_sample_) was measured against an ethanol blank. The absorbance of a negative control (A_control_) containing water was also measured. The ABTS radical scavenging activity was calculated by the following equation.

$$\text{ABTS scavenging activity }=\;\frac{{\text{A}}_{\text{control}}{\text{-A}}_{\text{sample}}}{{\text{A}}_{\text{control}}}\times \text{ 100\%}$$ (2)

### Cellular Antioxidant Activity (CAA) Assay

The CAA activity of PCG samples was measured as previously described [17]. Briefly, HepG2 cells were seeded into a 96-well microplate in growth medium and incubated at 37°C for 24 h. Growth medium was removed from the wells and the cells were washed with phosphate buffer solution (PBS). Then, two experimental protocols were performed: cells pretreated with PCG samples before 600 μM AAPH addition (PBS wash protocol) and or cells pretreated with simultaneously both PCG samples and AAPH (no PBS wash protocol). The EC_50_ of each sample was determined using fluorescent spectrometry (λ_excitation_ 485 nm and λ_excitation_ 535 nm), which was further converted to the CAA value (expressed as μmoles of quercetin equivalents per 100 g of the sample).

### Antiproliferative Assay

The antiproliferative assay was performed as previously described [18]. Briefly, the HepG2 cells were seeded in a 96-well microplate with growth medium and incubated at 37°C for 4 h. Growth medium was removed from the wells and different concentrations of the sample (dissolved in fresh medium) was added to the wells. Wells containing only growth medium was used as a negative control. After incubation at 37°C for 72 h, cell viability was determined using a previously developed methylene blue protocol, followed by EC_50_ value determination.

### α-Glucosidase Inhibition Assay

α-Glucosidase inhibition assays were performed as previously described [19]. Both α-glucosidase and pNP-G stock solutions were prepared using 0.1 M PBS at pH 6.9. 1 mL of α-glucosidase solution (0.2 U/mL) and 0.3 mL of the sample (1–5 μg/mL for PCG and 100–500 μg/mL for catechin) were mixed and incubated at 37°C for 10 min. After addition of 1 mL of 1 mM pNP-G solution, the mixture was incubated at 37°C for 20 min and 1 mL of anhydrous methanol then was added to stop the reaction. The absorbance of sample (A_sample_) at 405 nm was measured. The absorbance of PBS was measured as a negative control (A_control_). α-Glucosidase inhibition activity was calculated using the following equation.

$$\text{Enzyme inhibitory activity }=\;\frac{{\text{A}}_{\text{control}}{\text{-A}}_{\text{sample}}}{{\text{A}}_{\text{control}}}\times \text{ 100\%}$$ (3)

The inhibitory kinetics of α-glucosidase of each sample was measured using the Lineweaver-Burk equation. The pNP-G solutions (0.2–0.6 mM/L) were used as substrates for α-glucosidase. The PCG concentrations used in the reactions were set at as 0, 2 and 3 μg/mL, while the catechin concentrations were set at 0, 100 and 300 μg/mL.

### Statistical Analysis

The data were expressed as mean ± SD. Statistical comparisons were made by using the Student’s test. *P* <0.05 was considered to be significant.

## Results and Discussion

### PCG Synthesis and Structure

**Fig 2:**
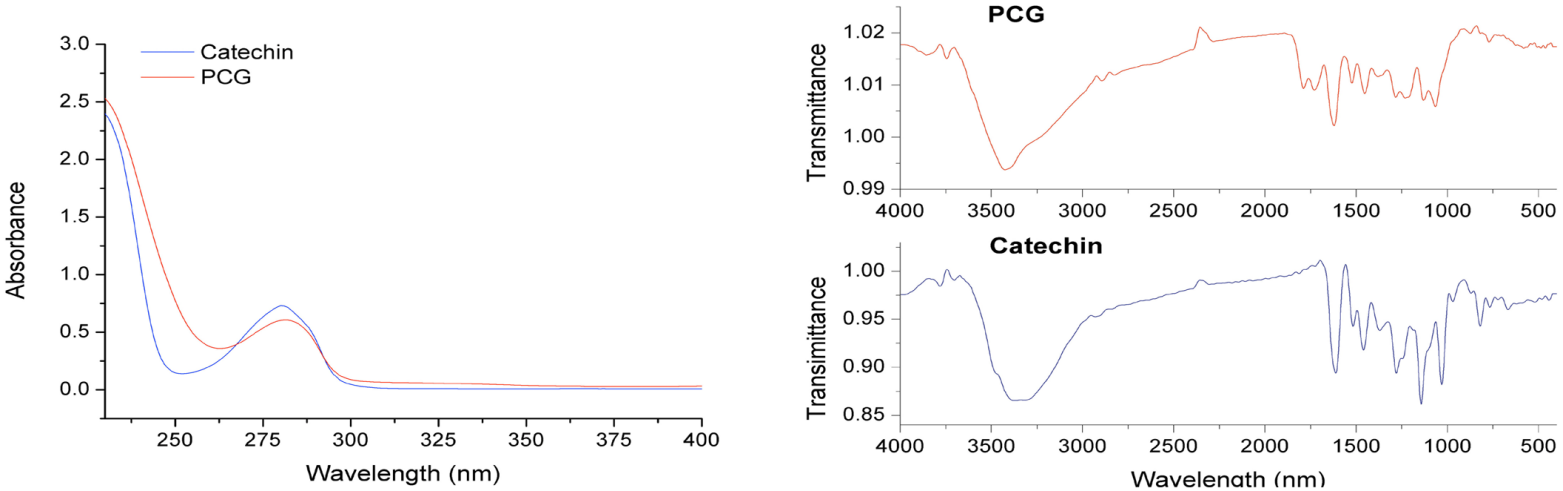
UV and IR spectra of catechin and PCG.

Previously, five catechin-aldehyde polycondensates were synthesized [12] and PCG had the highest aqueous solubility, as determined by the lack of PCG precipitate after the reaction completed. PCG is decorated with numerous carboxyl groups, which would allow for further modification to meet different demands. PCG was synthesized and the UV and IR spectra of both PCG and catechin were measured (Fig 2). Characteristic UV absorbance peaks for PCG and catechin were observed at 281 and 280 nm, respectively, which suggested that catechin moieties on PCG were retained. Characteristic IR peaks of catechin were observed at 3372 (phenolic hydroxyl group), 1613, 1518, 1460 (phenyl ring), 1144, and 1034 (C-O-C stretching vibration) cm^-1^. In addition, a new peak at 1789 cm^-1^ (carboxyl group) was observed by IR with PCG. PCG ^1^H and ^13^C NMR data were obtained, resulting in the following peaks: ^1^H NMR (DMSO-d_6_): δ = 0.9–1.5 (br, H-4), 3.5–4.2 (br, H-3), 4.3–5.5 (br, H-2 and 11), 6.2–7.0 (br, H-2’, 3’ and 6’), 8.2–9.2 (br, ArOH), 12–13 ppm (br, -COOH); ^13^C NMR (DMSO-d_6_): δ = 18 (C-4), 56 (C-11), 60 (C-3), 87 (C-2), 100–108 (C-6, 8 and 10), 113–117 (C-2’, 3’ and 6’), 130 (C-1’), 143–146 (C-4’ and 5’), 150–155 (C-5 and 9), 162 (C-7), 172 (-COOH). Based on NMR results, it was concluded that PCG formation resulted from an ethyl bridge between the C-6 and C-8 of catechin, coinciding with previous reports [12].

### DPPH and ABTS Radical Scavenging Activities

**Fig 3:**
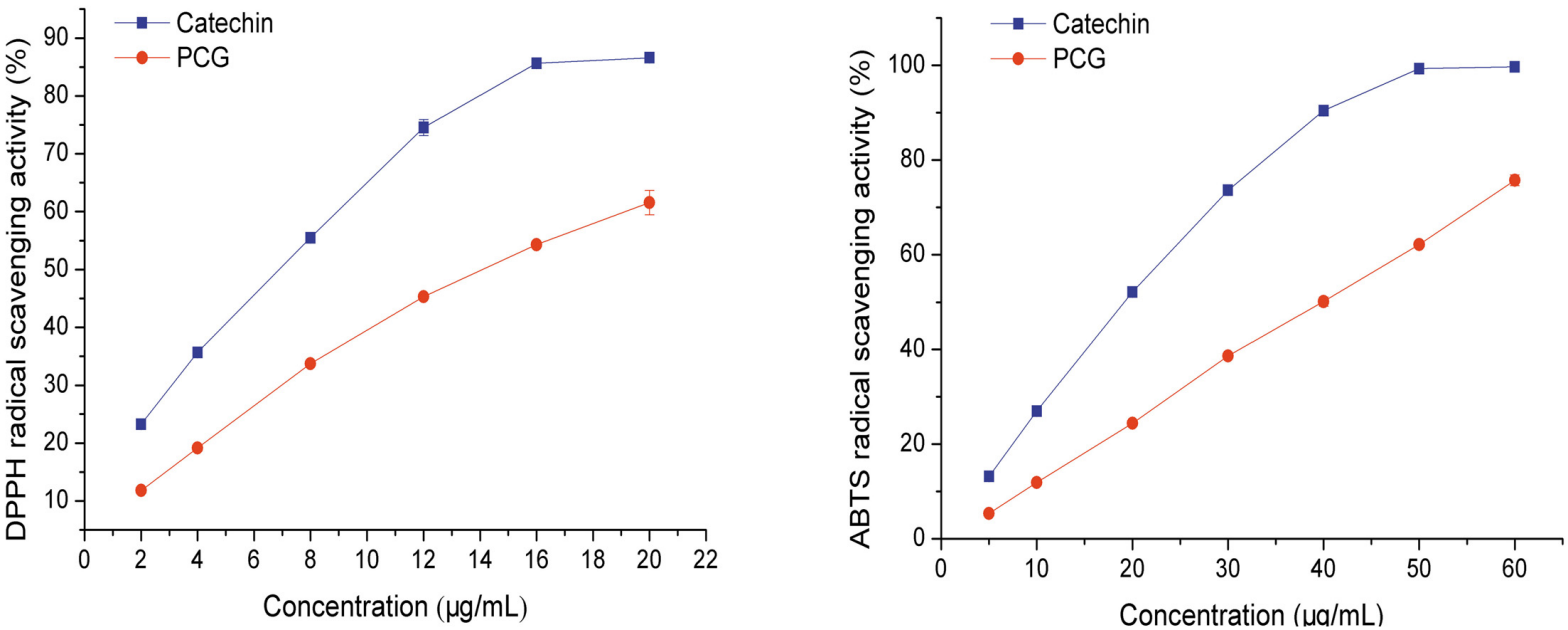
DPPH and ABTS radical scavenging activities of catechin and PCG.

The chemistry antioxidant activities of catechin and PCG were compared using DPPH and ABTS radical scavenging assays, which were sensitive enough to measure antioxidant activities at low sample concentrations over short time frames [20, 21]. Both catechin and PCG exhibited strong DPPH and ABTS radical scavenging activities in a dose-dependent manner (Fig 3). The IC_50_ values of catechin and PCG for DPPH radical scavenging activity were 5.98 and 14.25 μg/mL, respectively, while the ABTS radical scavenging activity IC_50_ values were 16.74 and 40.52 μg/mL, respectively. In summary, activities for catechin were superior to PCG on a per mass basis.

### Cellular Antioxidant Activity

**Fig 4:**
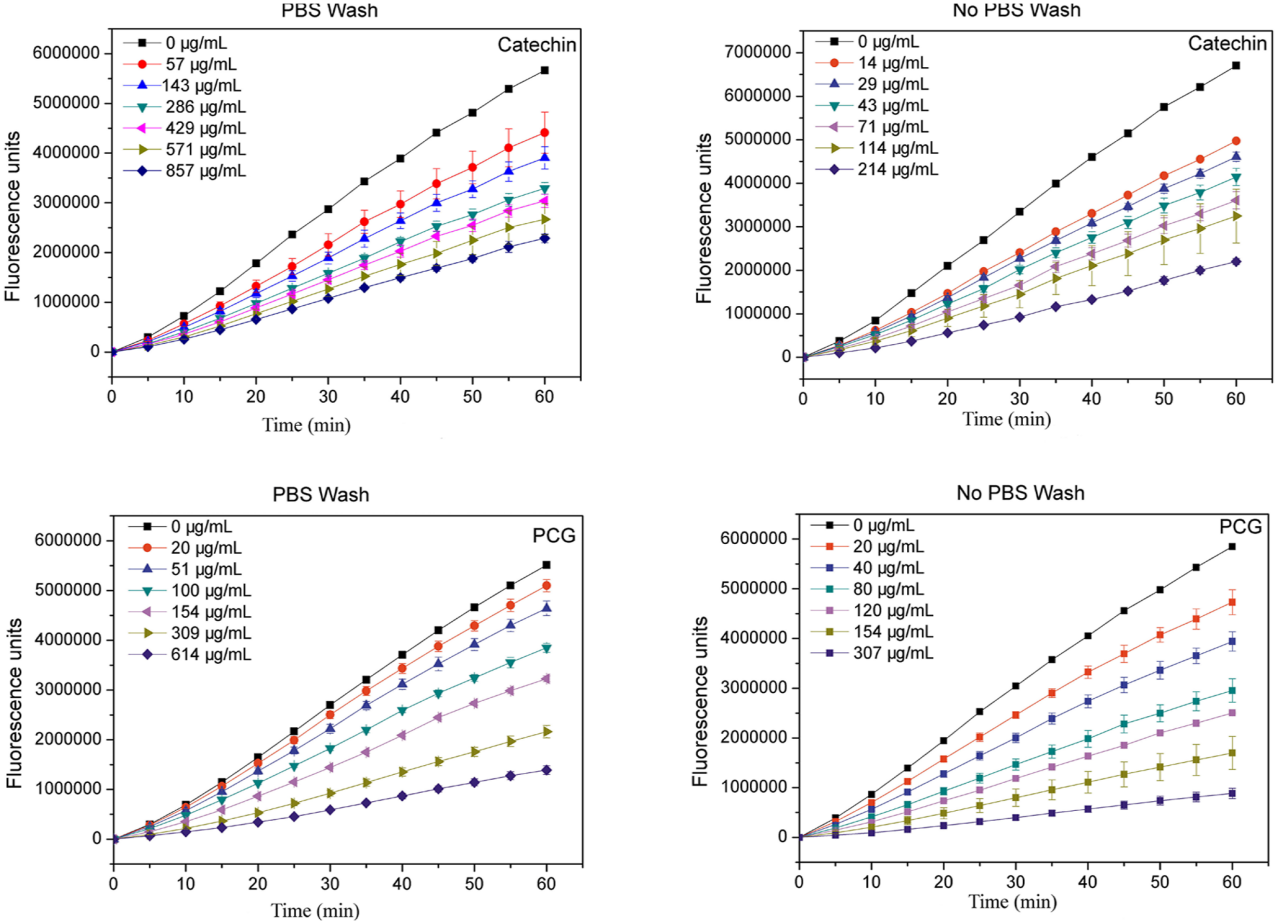
Peroxyl radical-induced oxidation of DCFH to DCF in HepG2 cells and the inhibition of oxidation over time by catechin and PCG.

The formation of excessive reactive oxygen species causes oxidative stress in the human body, which can lead to a variety of degenerative and chronic diseases—including cardiovascular diseases, type 2 diabetes, cancer, and Alzheimer's and Parkinson's Diseases [22]. Antioxidants can effectively reduce oxidative stress and current *in vitro* methods that measure antioxidant activity fail to reflect actual uptake, metabolism, and bioactivities in the body. Using a HepG2 cell model, an effective antioxidant CAA assay was established [23], and the effects of catechin and PCG on the peroxyl radical-induced oxidation of DCFH to DCF in cells were assessed (Fig 4). The enhancement in fluorescence from the formation of DCF was inhibited by catechin and PCG in a dose-dependent manner. The higher the fluorescence, the lower antioxidant activity of sample was. The calculated EC_50_ and CAA values for both the PBS and no PBS wash protocols are summarized in Table 1.

**Table 1:** Cellular antioxidant activity of catechin and PCG.

Extract	EC_50_ value (μg/mL)*		CAA value (μmol of quercetin / 100 g of sample)*	
	no PBS wash	PBS wash	no PBS wash	PBS wash
Catechin	73.13±6.47^a^	429.42±30.26^a^	18184.28±1616.81^a^	2244.63±155.77^a^
PCG	72.10±4.40^a^	200.26±2.80^b^	26695.08±1597.73^b^	7657.10±107.21^b^

*Values in the column with no letters in common are significantly different (p < 0.05).

When a PBS wash was adopted before ABAP treatments, the PBS would rinse out the samples that are loosely absorbed on the surface of the cell membrane. Lower EC_50_ or higher CAA values corresponded to stronger *in vivo* antioxidant activities, while differences in activity between catechin and PCG (no PBS wash) were not significant, suggesting that total antioxidant activities were similar between the two. The PBS wash protocol was used to reflect the intracellular antioxidant activity, and as the molecular weight and volume of PCG was higher than catechin, the cell membrane permeability of PCG should be lower than catechin. This would suggest that the CAA values for PCG using the PBS wash protocol should be lower than those for catechin, however the antioxidant activities were higher for PCG than catechin. This finding could be due to enhanced binding of PCG to the cell membrane as a result of the polymerization, which subsequently improved the cell protection effects.

### Antiproliferative Activity

**Fig 5:**
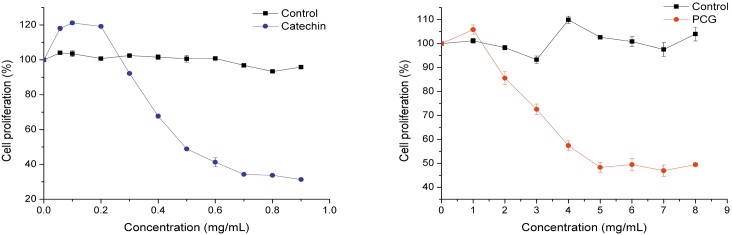
Percent inhibition of human HepG2 liver cancer cell proliferation by catechin and PCG.

Due to the number of liver cancer patients in the world, the HepG2 cell line has been widely adopted for numerous biochemical and medical studies. The antiproliferation effects of catechin and PCG were tested using a HepG2 cell and both samples inhibited HepG2 cell proliferation in a dose-dependent manner (Fig 5). The catechin had the strongest antiproliferative effects on HepG2 cells between 0.3–0.9 mg/mL, and treatment with 0.9 mg/mL catechin decreased HepG2 proliferation to 10% in comparison to the negative control. However, PCG demonstrated only weak antiproliferation effects on HepG2 cell, which could be attributed to poor membrane permeation due to its large molecular volume.

### α-Glucosidase Inhibitory Activity

**Fig 6:**
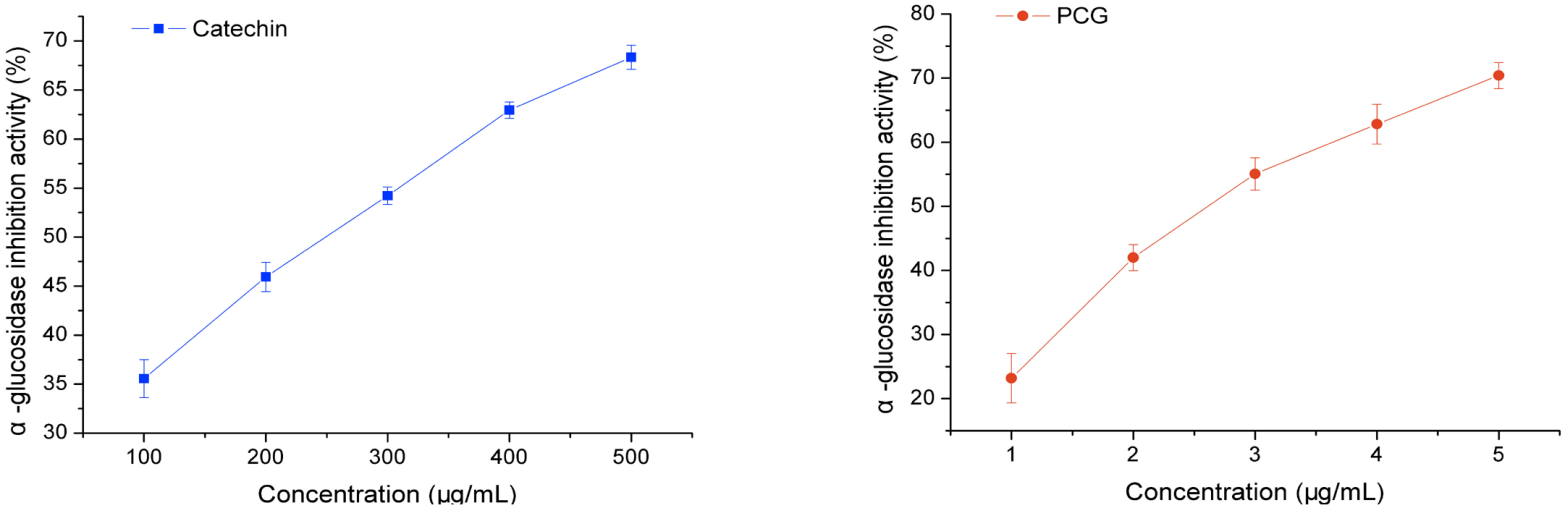
α-Glucosidase inhibitory activity of catechin and PCG.

**Fig 7:**
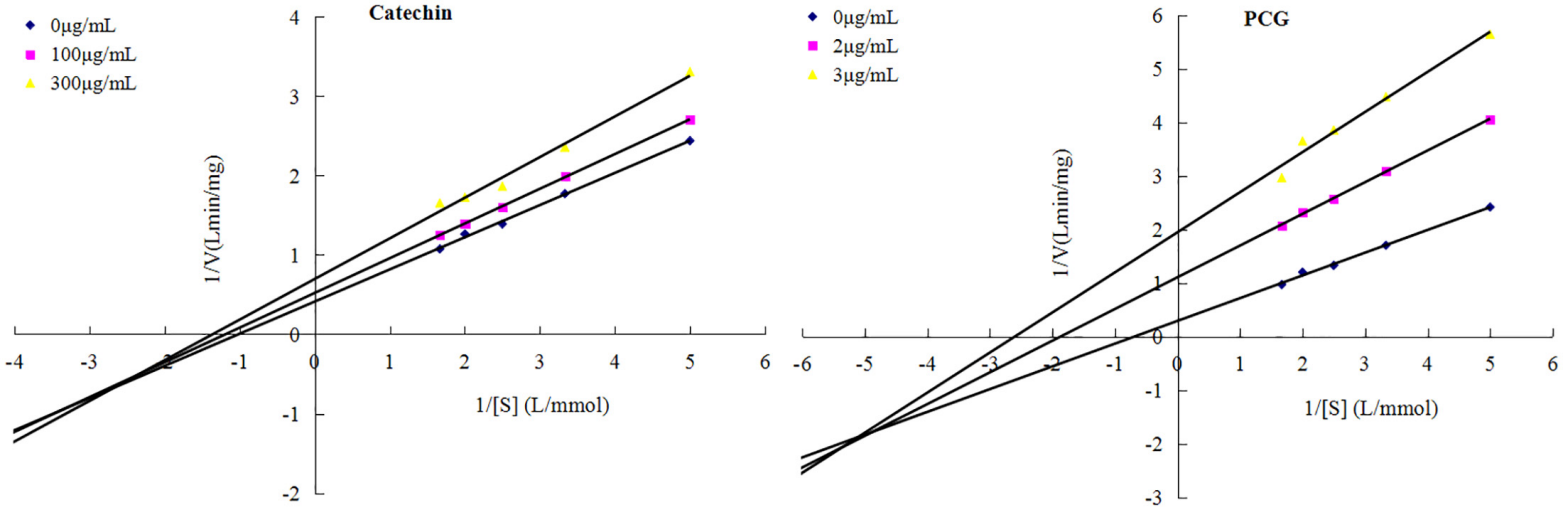
Lineweaver-Burk plots of the inhibition kinetics of catechin and PCG against α-glucosidase.

Diabetes mellitus is a common chronic disease that is characterized by hyperglycemia. Traditional therapies focus on controlling postprandial hyperglycemia using amylase and *α*-glucosidase inhibitors, which hinder the rapid absorption of carbohydrates by the body [24, 25], and *α*-glucosidase inhibitors have been to possess anti-viral and anti-HIV activities due the role of *α*-glucosidase in glycosidation of envelope glycoproteins [26]. Treatment with *α*-glucosidase inhibitors at >100 μg/mL resulted in negligible *α*-glucosidase inhibitory activity against catechin was observed (Fig 6). However, the inhibitor had marked *α*-glucosidase inhibitory activity against PCG in a dose-dependent manner. Compared to catechin (IC_50_ value, 239.27 μg/mL), the *α*-glucosidase inhibitory activity of PCG (IC_50_ value, 2.59 μg/mL) was increased 90-fold, which should be attributed to its strong enzyme protein binding capacity [27]. In order to investigate the inhibitory kinetics of catechin and PCG, the inhibition of activities were measured at varying concentrations of both *α*-glucosidase and inhibitor and the data was analyzed by Lineweaver-Burk plots (Fig 7). Together, the kinetics data indicated that both of the inhibitory behaviors were a mixture of noncompetitive and anticompetitive, which coincides with the *α*-glucosidase inhibitory regime of many flavonoid monomers [28]. This suggests that catechin and PCG inhibit activity by either binding the enzyme or the enzyme-substrate complex. In the previous reports, it was found that PCG could inhibit tyrosinase by binding the enzyme active site, respectively [7, 14]. The difference in enzyme structure could lead to the different inhibitory behaviors of PCG.

## Conclusions

Although the antioxidant and antiproliferative activities of PCG were inferior to those of monomeric catechin, PCG had much higher cellular antioxidant and *α*-glucosidase inhibitory activities than monomeric catechin. Polymerization significantly enhanced the binding capacity of catechin with both the cellular membrane and proteins, which led to an increase in corresponding bioactivities. As PCG synthesis is relatively simple and the molecule has strong bioactivities, PCG could be used as a nutraceutical to control postprandial hyperglycemia and offer antioxidant protection in functional food, medical and cosmetic industries.
